# Successful Surgical Treatment of Coronavirus Disease 2019 (COVID-19) Vaccination Related Upper Extremity Lymphedema: Case Report

**DOI:** 10.1055/a-2448-3403

**Published:** 2025-07-08

**Authors:** Hyung Hwa Jeong, Dong Jin Kim, Hyunsuk Peter Suh, Chang Sik John Pak, Joon Pio Hong

**Affiliations:** 1 Department of Plastic and Reconstructive Surgery, Hanyang University, College of Medicine, Seoul, Korea; 2 Department of Plastic and Reconstructive Surgery, University of Ulsan, College of Medicine, Seoul Asan Medical Center, Seoul, Korea

**Keywords:** COVID-19 vaccines, adverse effect, lymphedema, secondary lymphedema, surgical procedures

## Abstract

Lymphedema is rare adverse effect of coronavirus disease 2019 (COVID-19) vaccination that has been reported in several studies. We present a case of surgically treated secondary lymphedema after COVID-19 vaccination. The patient presented lymphedema at the upper extremity with no specific history except the COVID-19 vaccination 18 months before the visit. Lymphaticovenous anastomosis and liposuction were performed on the posterolateral aspect of the forearm and the upper arm. The volume of the affected arm was reduced to more than 54% at 8 months postoperatively. With precise surgical planning, secondary lymphedema resulting from COVID-19 vaccination could be successfully treated surgically.

## Introduction

After the outbreak of coronavirus disease 2019 (COVID-19), millions of doses of COVID-10 vaccine were administrated globally. Among them, more than 2,500 million doses of the BNT162b2 (Pfizer Inc., New York, NY, United States and BioNTech, Mainz, Germany) vaccine were administrated until 2021. 1 Lymphedema is a rare adverse effect of COVID-19 vaccination, which was reported in several studies. Both lower extremity and upper extremity lymphedemas were reported. 2 3 4 Furthermore, outbreaks of lymphedema after COVID-19 vaccination in breast cancer patients and frequent lower leg cellulitis after vaccination have also been reported. 5 6 In summary, although its incidence is relatively rare, lymphedema after COVID-19 vaccine certainly exists, and treatment option is not reported well.

Lymphedema is a condition caused by mechanical or intrinsic disruption of lymphatic flow. Surgical treatment options for the lymphedema are divided into two categories: ablative and physiologic surgeries. Ablative surgery can be represented by the Charles procedure and liposuction, which remove hypertrophied fat tissue and fibrosis induced by the chronic condition of lymphedema. Physiologic surgery includes lymphaticovenous anastomosis (LVA), lymph node-to-vein anastomosis (LNVA), and vascularized lymph node transfer (VLNT). Physiologic surgery nowadays plays the main role in surgical treatment of lymphedema. However, in advanced stage of lymphedema, excess adipose tissue is hard to be removed with physiologic surgery. 7 In those cases, liposuction is reported to be effective in reducing the volume and improving the quality of life. 8

In this case report, we present a case of secondary lymphedema that occurred after the vaccination with the Pfizer (BNT162b2) vaccine including the booster dose. The condition was successfully treated with a combination of physiologic surgery (LVA) and ablative surgery (liposuction).

## Case

Informed consent was obtained from the patient for the publication of this case report and any accompanying images. The patient was thoroughly informed about the purpose of the report, the nature of the information to be disclosed, and the potential implications of its publication. The patient understood their participation was voluntary and had the right to withdraw consent at any time without any impact on their medical care. The patient provided written consent, agreeing to share their medical case for educational and research purposes.

**Fig. 1:**
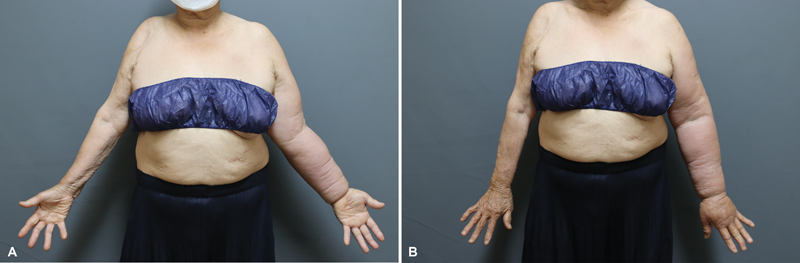
( **A** , **B** ) Preoperative findings. Ext., extremity; Inj., injection; Lt., left; Rt., right.

An 80-year-old female patient presented to the plastic surgery clinic with right upper extremity edema with pitting and firm change on lateral and posterior aspects of the upper arm and the forearm ( Fig. 1 ). There was no history of trauma at the affected side of the arm and trunk nor the operative history. The patient got vaccination for COVID-19 18 months before the visit. It was the secondary vaccination with BNT162b2 (Pfizer-BioNTech), and edema occurred on the same side of the arm where the vaccine was administrated. The edema started 3 days after vaccination. The patient had multiple events of cellulitis and received intravenous antibiotics. Then the patient was referred to the rehabilitation medicine department and received compression treatment using compression bandage, which was prescribed from the rehabilitation medicine department for 1 year. However, response to the treatment was refractory. Then the patient was referred to the plastic surgery department. The patient got admission from the Ministry of the Health and Welfare of Korea about the relationship between edema and vaccination as an adverse effect.

**Fig. 2:**
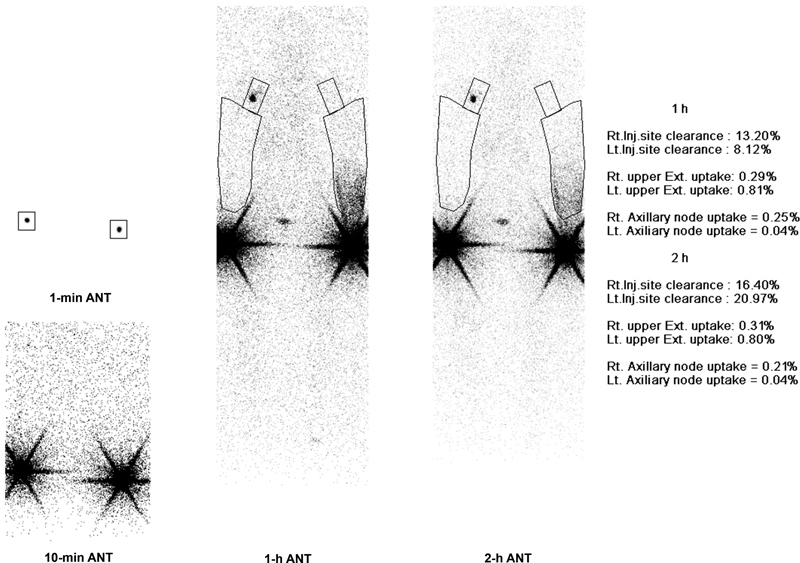
Preoperative lymphoscintigraphy.

To exclude the possible cause of the lymphedema, chest computed tomography (CT) was taken; however, there was no evidence of any malignancy. As a preoperative workup, upper extremity lymphoscintigraphy and magnetic resonance lymphangiography (MRL) were taken. In lymphoscintigraphy, increased dermal backflow at the distal forearm of the affected side and absent uptake at the ipsilateral axillary lymph node was found, which was correlated with signs of lymphedema ( Fig. 2 ). MRL revealed multiple dilated lymphatic vessels surrounding the left wrist and extending to the forearm along with diffuse dermal backflow at the dorsum of the hand and mid-forearm. The circumferential size of the patient and bioelectric impedance test are described in Table 1 .

**Table 1:** Circumference and bioimpedance analysis data of the patient (cm, ± cubital fossa)

	BMI	BIA a	+ 15	+ 10	+ 5	−5	−10	−15	Volume (cm ^3^ )
**Pre-op**	**27.0**								
Affected		5.23	33.5	34.0	35.0	32.0	30.3	28.3	2,285.98
Normal		1.99	27.0	23.5	20.0	20.5	17.5	15.0	1,082.01
**Post-op 3 mo**	**25.6**								
Affected		3.09	28.0	27.0	26.0	23.5	22.0	19.0	1,335.19
Normal		2.16	26.0	23.0	21.0	21.0	17.5	15.5	1,050.36
**Post-op 8 mo**	**25.6**								
Affected		2.98	25.5	24.0	22.5	22.5	22.0	20.0	1,242.24
Normal		1.94	26.0	23.0	21.0	21.5	17.5	15.5	1,050.36

a Bioelectric impedance analysis: extracellular water/total body water.

**Fig. 3:**
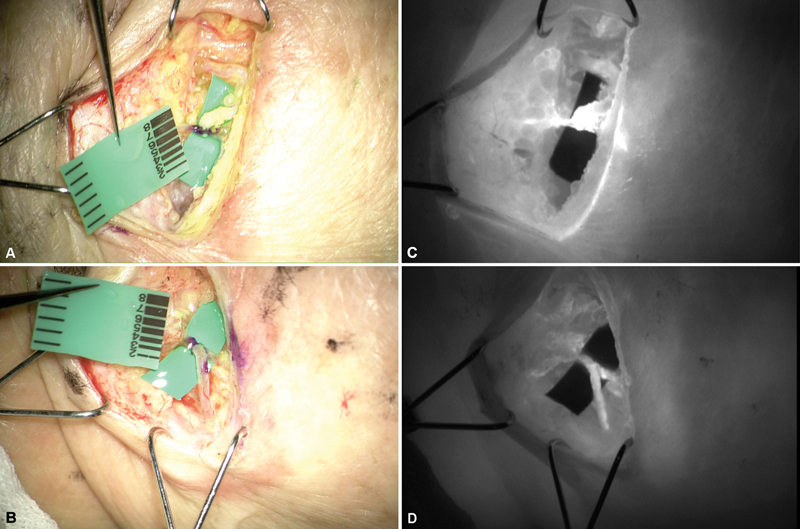
( **A, B** ) The picture after the lymphaticovenous anastomosis. ( **C, D** ) Indocyanine green lymphography of each anastomosis site.

For the patient, as there was no operative history on the axillary area, LVA at the wrist level and liposuction for the posterolateral aspect of the forearm and the upper arm were planned. Before the operation, the lymphatic vessel was traced using ultrasonography and indocyanine green (ICG) lymphangiography. Then the incision site was determined according to the preoperative tracing. At two incisions on the wrist, two ectatic lymphatic vessels within thin walls were found, measuring 0.4 and 0.6 mm. Successful LVA was performed in a side-to-end manner. After the anastomosis, ICG washout to the anastomosed vein could be seen ( Fig. 3 ). Then, ultrasound-assisted liposuction was performed using the tumescent technique, resulting in the removal of a total of 850 mL of the fibrotic tissue and fat. After operation, immediate compression was applied with a double-layered compression bandage (Deflate, HS Healing Solution Limited, Tsimshatsui, Hong Kong).

**Fig. 4:**
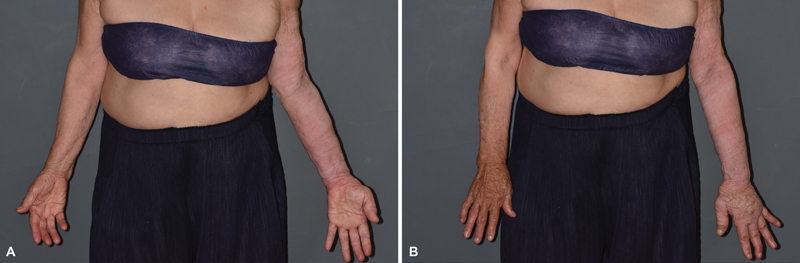
( **A, B** ) Eight-month postoperative findings.

**Fig. 5:**
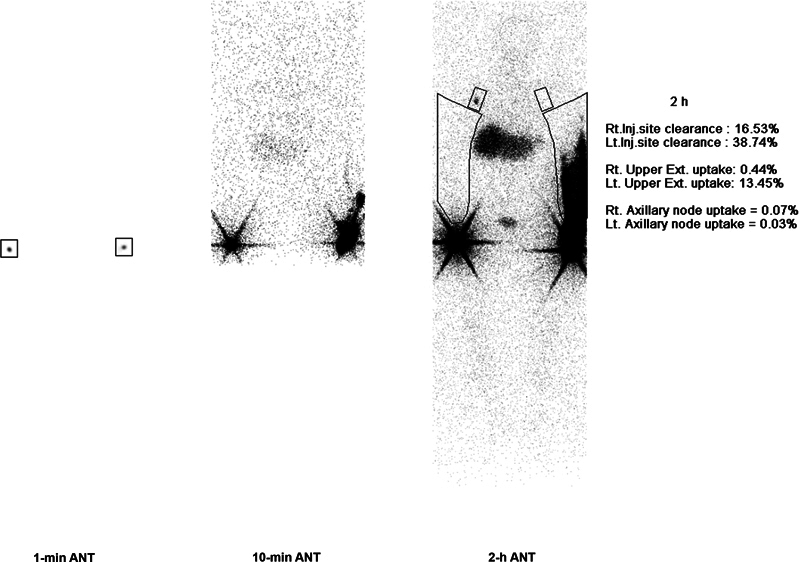
Postoperative lymphoscintigraphy (8 months post-op). ANT, anterior; Ext., extremity; Inj., injection; Lt., left; Rt., right.

The circumference of the affected arm was reduced by 8 cm at the upper arm and 8.3 cm at the distal forearm at 8 months of follow-up. The estimated arm volume was reduced from 2,285.98 to 1,242.24 cm ^3^ (reduction rate 54.34%; Fig. 4 ). The bioelectric impedance analysis of extracellular fluid of the affected limb was reduced from 5.23 to 2.98. Fig. 5 shows the lymphoscintigraphy findings at 8 months of follow-up. The patient continued the compression therapy using a compression bandage until the last follow-up.

## Discussion

The cause-and-effect relationship of upper extremity lymphedema and COVID-19 vaccination should be carefully judged. There are limited reports of secondary lymphedema occurring after COVD-19 vaccination. Interestingly, there are numerous reports of lower extremity lymphedema, but there is only one report of upper extremity lymphedema. The case report of upper extremity lymphedema documented the development of edema following the Pfizer-BioNTech mRNA vaccine booster, which is the same as our case.

Vaccine-induced lymphadenopathy, resulting from antigen transmission to the lymph node, is suggested as a pathophysiology of lymphedema following vaccination. 9 According to the literature, lymphadenopathy caused by vaccination can aggravate lymphatic drainage, especially in vulnerable patients, which can lead to the development of lymphedema. Furthermore, some studies suggest that the COVID-19 mRNA vaccination may be linked to an inflammatory response to hyaluronan. 10 11 Lymphatics are the primary pathway for hyaluronan drainage, of which dysfunction may result in hyaluronan accumulation. 12 These may be the possible mechanism of mRNA COVID-19-vaccine-induced lymphedema. Several studies reported lymphedema and cellulitis following COVID-19 vaccinations. 3 5 However, there are no reports of successful treatment of secondary lymphedema after COVID-19 vaccination, either surgically or nonsurgically. In this case, considering the previous failure of physical treatment including compression, surgical treatment was attempted. With meticulous preoperative workup and planning, successful treatment could be achieved.

In the follow-up lymphoscintigraphy, overall dermal backflow was increased compared to the preoperative study. There are some debates about the dermal backflow pattern in lymphoscintigraphy. Dermal backflow was deemed a sign of lymphedema; however, its absence is considered backwardly, as a sign of most advanced stage of lymphedema. 13 Furthermore, there is report that the surgical outcome of lymphedema is better in the cases with dermal backflow at lymphoscintigraphy compared with the cases without dermal backflow. 14 According to a study, treatment decisions should be based on both the clinical symptom and the severity of dermal backflow. 15 Therefore, we conclude that increased dermal backflow after surgery can be a favorable sign of clinical improvement.

It is interesting to note that edema in the affected hand dorsum and fingers was significantly reduced with prominent wrinkle even if the operation including liposuction was not performed on the hand dorsum. This finding indicates that surgical management of lymphedema can not only benefit the operated region but also improve overall lymphatic washout throughout the whole affected limb. However, as lymphedema is a deteriorating condition, surgical options should be provided to the patients along with decongestive treatment.

### Conclusion

Lymphedema is a rare side effect of COVID-19 vaccination with limited treatment options. With precise surgical planning, secondary lymphedema that occurred after COVID-19 vaccination could be successfully treated surgically.
